# Commentary to: Lymphocytic UV autofluorescence: A novel UV-induced fluorescence dermoscopy finding in lichen nitidus: A series of two cases

**DOI:** 10.1016/j.jdcr.2025.11.048

**Published:** 2025-12-12

**Authors:** Paweł Pietkiewicz, Marian Voloshynovych, Julien Anriot, Cristian Navarrete-Dechent

**Affiliations:** a Prisca Sapientia Institute, Zurich, Switzerland; b Zwierzyniecka Medical Center, Poznań, Poland; c Department of Dermatology and Venereology, Ivano-Frankivsk National Medical University, Ivano-Frankivsk, Ukraine; d Lux Skin, Ivano-Frankivsk, Ukraine; e Claude Bernard Lyon-1 University, Lyon, France; f Department of Dermatology, Centre Léon Bérard, Lyon, France; g Melanoma and Skin Cancer Unit, Escuela de Medicina, Pontificia Universidad Católica de Chile, Santiago, Chile

*To the Editor:* We read with interest the case report by Varun et al^1^ published in the November issue of *JAAD Case Reports*, describing lymphocytic UV autofluorescence in generalized lichen nitidus. The authors correlated clinical, dermatoscopic, UV-induced fluorescence dermoscopy (UVFD), and histopathologic findings in 2 pediatric cases. Both demonstrated well-defined hypopigmented clods with occasional peripheral scale and brownish dots that exhibited blue-white fluorescence under UVFD. The authors attributed this fluorescence to autofluorescence of the dermal lymphocytic infiltrate, referencing studies using fluorescence microscopy,^2^ spectrophotometric microscopy,^3^ and flow cytometry.^4^

Although the proposed mechanism is intriguing, we note that other conditions with dense lymphocytic infiltrates—such as lichen planus or folliculotropic mycosis fungoides—do not typically display blue fluorescence on UVFD in our experience. We, therefore, suggest considering an alternative fluorophore that was not discussed: subtle serous crusts rich in bilirubin. Brown dots may represent healing erosions that produce blue-to-green fluorescence depending on their stage and sun exposure.^5^ Such subtle erosions may result from intentional scratching or friction over lichen nitidus papules by both young patients, leading to minimal exudation that can be inconspicuous on conventional dermoscopy but nonetheless fluoresces under UVFD (Fig 1).

**Fig 1:**
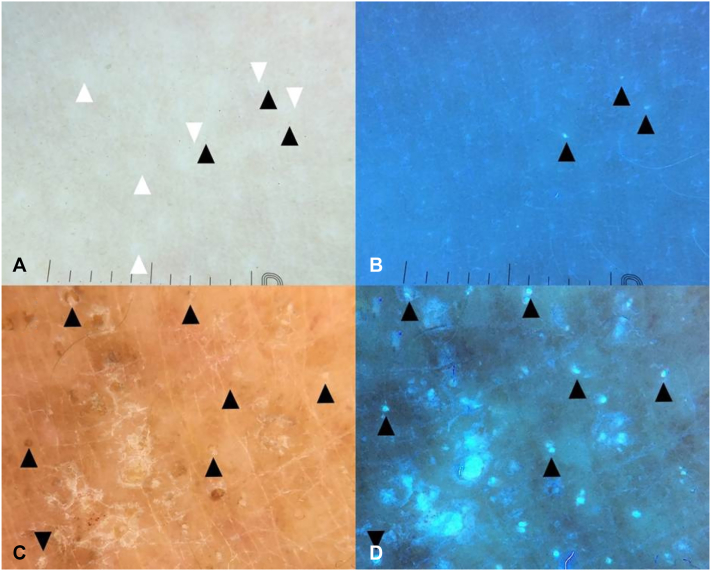
**A,** Polarized dermoscopy of a localized lichen nitidus in a young boy featuring subtle tan clods (serous erosions; *black arrowheads*) occasionally superimposed over white clods of parakeratosis/acanthosis (*white arrowheads*). **B,** UV-induced fluorescence dermoscopy of lichen nitidus demonstrates bright bluish clods corresponding to serous erosions (*black arrowheads*). **C,** Polarized dermoscopy of eczematous dermatitis in elderly woman featuring brown, orange and tan serokeratotic crusts (*black arrowheads*). **D,** UV-induced fluorescence dermoscopy of eczematous dermatitis in elderly woman demonstrating blue-greenish fluorescence of the areas corresponding to serokeratotic crusts (*black arrowheads*).

We commend the authors for highlighting these uncommon presentations and hope this additional consideration supports future interpretation of UVFD features in lichen nitidus and related dermatoses.

Sincerely,

Paweł Pietkiewicz (on behalf of all authors)

## Conflicts of interest

None disclosed.
